# Treatment of Pregnant Women With Fear of Childbirth Using EMDR Therapy: Results of a Multi-Center Randomized Controlled Trial

**DOI:** 10.3389/fpsyt.2021.798249

**Published:** 2022-02-10

**Authors:** M. A. M. Baas, M. G. van Pampus, C. A. I. Stramrood, L. M. Dijksman, J. W. Vanhommerig, A. de Jongh

**Affiliations:** ^1^Department of Obstetrics and Gynecology, OLVG, Amsterdam, Netherlands; ^2^Department of Quality and Safety, St. Antonius Hospital, Nieuwegein, Netherlands; ^3^Department of Research and Epidemiology, OLVG, Amsterdam, Netherlands; ^4^Academic Centre for Dentistry Amsterdam (ACTA), University of Amsterdam, Vrije Universiteit University Amsterdam, Amsterdam, Netherlands; ^5^PSYTREC, Bilthoven, Netherlands; ^6^School of Health Sciences, Salford University, Manchester, United Kingdom; ^7^Institute of Health and Society, University of Worcester, Worcester, United Kingdom; ^8^School of Psychology, Queen's University, Belfast, United Kingdom

**Keywords:** fear of childbirth, tocophobia, eye movement desensitization and reprocessing therapy, pregnancy, childbirth, treatment, EMDR

## Abstract

Fear of childbirth (FoC) occurs in 7. 5% of pregnant women and has been associated with adverse feto-maternal outcomes. Eye Movement Desensitization and Reprocessing (EMDR) therapy has proven to be effective in the treatment of posttraumatic stress disorder (PTSD) and anxiety; however, its effectiveness regarding FoC has not yet been established. The aim was to determine the safety and effectiveness of EMDR therapy for pregnant women with FoC. This single-blind RCT (the OptiMUM-study, www.trialregister.nl, NTR5122) was conducted in the Netherlands. FoC was defined as a score ≥85 on the Wijma Delivery Expectations Questionnaire (WDEQ-A). Pregnant women with FoC and a gestational age between 8 and 20 weeks were randomly assigned to EMDR therapy or care-as-usual (CAU). The severity of FoC was assessed using the WDEQ-A. Safety was indexed as worsening of FoC symptoms, dropout, serious adverse events, or increased suicide risk. We used linear mixed model analyses to compare groups. A total of 141 women were randomized (EMDR *n* = 70; CAU *n* = 71). No differences between groups were found regarding safety. Both groups showed a very large (EMDR *d* = 1.36) or large (CAU *d* = 0.89) reduction of FoC symptoms with a mean decrease of 25.6 (EMDR) and 17.4 (CAU) points in WDEQ-A sum score. No significant difference between both groups was found (*p* = 0.83). At posttreatment, 72.4% (EMDR) vs. 59.6% (CAU) no longer met the criteria for FoC. In conclusion, the results are supportive of EMDR therapy as a safe and effective treatment of FoC during pregnancy, albeit without significant beneficial effects of EMDR therapy over and above those of CAU. Therefore, the current study results do not justify implementation of EMDR therapy as an additional treatment in this particular setting.

## Introduction

Severe fear of childbirth (FoC), sometimes also referred to as tocophobia (1), affects a relevant proportion of pregnant women (7.5%) (2) and has been found to be associated with adverse maternal and neonatal outcomes. FoC occurs both in women who did not give birth before (nulliparous; primary FoC) and in women with at least one previous childbirth (multiparous; secondary FoC). Except for a few studies (3, 4), most studies include women regardless of parity, and make no distinction between primary and secondary FoC. Evidence suggests that during pregnancy, FoC may negatively affect the experience of pregnancy and the transition to parenthood (5). Occasionally, women deny pregnancy and avoid prenatal care, or even decide to terminate their pregnancy (6). Also, some women with FoC request a cesarean section (7, 8). Furthermore, FoC predisposes women to a negative delivery experience (9), a six-fold increased risk of developing childbirth-related posttraumatic stress disorder (PTSD) (10, 11) and an increased risk of postpartum depression (12). In addition, elevated maternal stress during pregnancy has been associated with negative outcomes such as low birth weight (13) and preterm birth (14).

Several previous studies aimed at determining the effectiveness of interventions for FoC revealed positive treatment outcomes. For example, a randomized controlled trial (RCT) investigating the effects of telephone psycho-education (*n* = 170) by midwives showed a significant reduction of FoC compared to care-as-usual (*n* = 169) (15). Likewise, an RCT comparing five weekly sessions of motivational interviewing to care-as-usual with a total of 70 women demonstrated significant improvement in the intervention group regarding percentage of women who scored 60 or lower on the WDEQ-A, and a decline in FoC sum score (16). Another RCT that compared group psycho-education with relaxation or conventional care among 371 women found that psycho-education was associated with a significantly more positive birth experience (WDEQ-B), decreased postpartum depressive symptoms, and more uncomplicated vaginal deliveries than conventional care (3, 4). Lastly, a study among 134 women who were randomly allocated to either haptotherapy, psycho-education *via* internet, or care-as-usual, showed haptotherapy to be associated with a significant decrease of FoC (17). However, not all studies showed a significant positive treatment outcome for interventions aimed at reducing FoC. An RCT investigating cognitive therapy added to routine antenatal care among 176 pregnant women did not result in a significant reduction of anxiety, or reduction of requests for elective cesarean sections (18).

It is important to note that adverse effects in women who underwent treatment for FoC have also been described. The results of a non-randomized trial evaluating counseling by specialized midwives, in which women were encouraged to talk about the nature of their fear and previous traumatic childbirth experiences, showed that after 1–14 counseling sessions (mean 4) a significant higher percentage of women experienced a negative childbirth experience and posttraumatic stress symptoms (19). Another study found that attending a program for childbirth-related fear was associated with an increased likelihood of cesarean section or a negative birth experience, and were significantly more often fearful 1 year after giving birth compared to no counseling (20). More recently, an RCT comparing cognitive therapy to care-as-usual in 282 women showed significantly higher depression and anxiety scores mid-pregnancy after cognitive therapy compared to care-as-usual (21). Hence, although there are promising results in some studies, research to determine safety and effectiveness of treatment for pregnant women with FoC is still needed.

Eye movement desensitization and reprocessing (EMDR; for a description: https://www.emdria.org/about-emdr-therapy/) therapy (22) is a recommended treatment for PTSD (23, 24). Several case studies and case series found preliminary evidence regarding the safety and effectiveness of EMDR therapy for treating pregnant women diagnosed with PTSD (25–27). EMDR therapy has also been found to be capable of processing memories of distressing events in a wide variety of other conditions (28) including traumatic triggers of the vomiting in pregnant women with hyperemesis gravidarum (29), which is a pregnancy condition with severe nausea, vomiting, weight loss, and dehydration. A recent review concluded that given “the fact that so far no adverse effects on the unborn child have been reported associated with the application of trauma-focused therapy, treatment of PTSD during pregnancy is most likely safe” (25). However, evidence from randomized controlled trials supporting this claim are still lacking; this not only holds true for PTSD, but also for FoC.

The purpose of the present study was to determine the safety and effectiveness of EMDR therapy for pregnant women with FoC. Regarding the safety of the intervention, we hypothesized that EMDR therapy would not be associated with a significant increase of symptoms of FoC, more serious adverse events, suicide risk, or a larger dropout compared to care-as-usual (CAU). With regard to its effectiveness, we hypothesized that EMDR therapy would result in a significantly larger symptom reduction compared to CAU. Moreover, we hypothesized that EMDR therapy would result in a significantly lower percentage of FoC diagnoses compared to CAU at follow-up measurements at 32 weeks gestational age.

## Materials and Methods

### Design

The OptiMUM study was a single-blinded multi-center RCT of which the study design has been published previously (30). The study included two separate RCTs with overlapping design, which differed in diagnosis of the included participants. The results of the first RCT concerning pregnant women with PTSD after previous childbirth has been described as a systematic review and case study elsewhere (25). The present paper pertains to the RCT concerning pregnant women with FoC. Our registered protocol also included obstetrical outcomes. These outcomes are not included in the current article, but will be reported in a future publication. The OptiMUM study was approved by the Medical Research Ethics Committee, and prospectively registered in the Dutch trial register (www.trialregister.nl, NTR5122).

### Participants

Pregnant women were recruited during their antenatal consultations in two teaching hospitals and five community midwifery practices in the Netherlands between February 2015 and November 2019. All pregnant women with a gestational age between 8 and 20 weeks were invited to participate in the screening. Inclusion criteria were a sum score on the WDEQ-A of ≥85 (31, 32). Exclusion criteria were age <18 years old, current psychological treatment, and the presence of a childbirth-related PTSD according to DSM-5 (33). After trial commencement, an addition to the exclusion criteria was made for women with intermediate or high suicide risk, or severe psychotic disorder [based on the Mini international neuropsychiatric interview (MINI)-plus] (34), since these psychiatric comorbidities would not be in accordance with our low-risk study design.

### Procedure

During routine antenatal checks, all eligible pregnant women with a gestational age between 8 and 20 weeks were screened for FoC using the WDEQ-A (35). Women with FoC were invited for a clinical interview during which psychological comorbidity was assessed using the MINI-plus (34) and Clinician-administered PTSD scale (CAPS-5) (36), and exclusion criteria were checked. When eligible, women were randomized to the treatment group or care-as-usual group on a 1:1 ratio by block randomization with random block size using an independent computer program (37). Stratification variables were not applicable.

In the EMDR therapy group, measurements of FoC took place at the start of each therapy session. Follow-up measurements were evaluated by a blinded assessor with a second clinical interview at 32–34 weeks of gestational age (T1). Two to three months after childbirth, an email questionnaire was sent, including self-report questionnaires and questions about possible support/treatment for FoC that patients arranged themselves.

### Intervention

EMDR therapy group; in addition to standard antenatal care, the participants received (a maximum of) three 90-min sessions of EMDR therapy by a psychologist. All seven participating psychologists had 2 years additional training as a clinical psychologist, completed the accredited basic and advanced EMDR training course, and had at least 1 year experience with providing EMDR therapy. Measurement of FoC symptoms took place at the start of each session. All therapy sessions were videotaped to allow for rating of treatment fidelity and for supervision purposes.

In the OptiMUM-study, EMDR therapy included the EMDR standard Dutch protocol (22, 38). The procedure applied in the present study a three-pronged approach of processing (1) disturbing memories of *past* experiences that caused the fear; (2) catastrophic images fuelling the anticipatory fear such as the baby dying during childbirth, or having unbearable pain (i.e., flashforwards) (39); and (3) triggers that *currently* evoke distress (using Shapiro's “mental video check”) (22). EMDR therapy was carried out with the use of rapid deployment of sets of eye movements. To maximize taxation of patients' working memory if needed, the therapist was allowed to add working memory taxation by using faster eye movements, change directions of the movements, or the use of extra earphones with a clicking sound, or hand-holdable pulsers providing alternating tactile stimulation memory (28).

In the CAU group, participants received standard antenatal care with routine obstetrical checks provided by their hospital or midwifery practice. EMDR therapy or other interventions aimed at reducing FoC were not routinely offered. Assuming good clinical care, healthcare providers might refer anxious or traumatized pregnant women according to their own clinical practices and guidelines. Type and frequency of any form of professional care was registered for both groups.

### Measures

The severity of FoC was measured by the WDEQ-A. The WDEQ (40) is a 33-item self-report questionnaire with two versions: assessing antenatal FoC (WDEQ-A) or childbirth experience (WDEQ-B). It was designed monofactorial with a sum score between 0 and 165, but can be used dichotomous with a cutoff of ≥85 (31, 32). Safety was assessed by measuring the occurrence of worsening of FoC symptoms, dropout (i.e., not starting EMDR treatment, or not completing treatment after one or more previous EMDR sessions), serious adverse events, and increased suicide risk (i.e., proportion high suicide risk based on MINI-plus).

Psychological comorbidity was assessed with a clinical interview, including the Dutch versions of the MINI-plus (34) and CAPS-5 (36).

### Statistical Methods

Before starting analyses, all data were screened for data-entry errors and outliers. The extent of missing data was assessed and assumptions for the analyses were checked. To compare groups at baseline measurements, *p*-values were calculated with 2-tailed independent Mann–Whitney *U* test (for continuous variables) and Fisher's exact test (for categorical variables) to compare groups. All analyses were performed intention-to-treat. The sample size was calculated at 51 participants in each group to obtain a power of 80% to detect an effect size of 0.5 (30). The WDEQ-A sum scores were modeled using linear mixed models (LMM) to adjust for repeated measurements. Time was used as a categorical variable (number of visits), and treatment condition was used as a fixed effect. Within-group effect sizes were calculated with Cohen's *d* (41). Clinically relevant symptom change on the WDEQ-A was assessed by reporting the Reliable Change of symptoms (RC) (42, 43) and the percentage of women with a WDEQ-A sum score below the cutoff of ≥85. The RC of symptoms was calculated for each group to adjust for possible measurement errors, and resulted in symptom improvement and worsening scores above 5.3 (CAU) or 4.9 (EMDR) on WDEQ-A. Our study was not powered for measures indexing safety; therefore, these outcomes were shown descriptively. The level of significance was set at α = 0.05. Analyses were performed using IBM SPSS Statistics for Windows, version 23.0.

## Results

**Figure 1:**
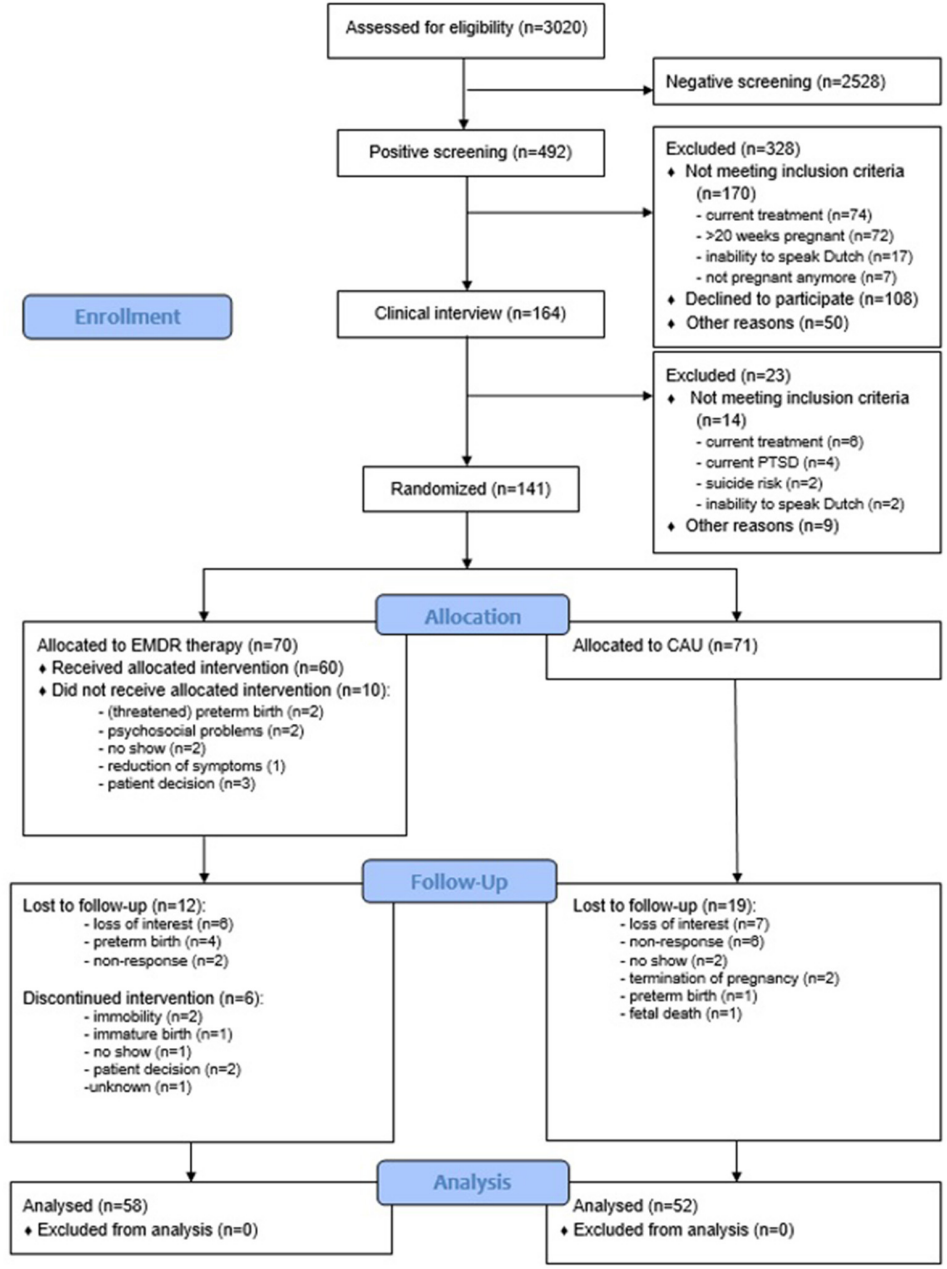
Flow diagram. CAU, CAre-as-Usual; EMDR, Eye Movement Desensitization and Reprocessing therapy.

In total, 141 pregnant women were included, of which 110 completed the post-treatment measurement. Figure 1 shows the patient flow through the trial. At T0, no statistically significant differences were found between the EMDR therapy and the CAU group with respect to demographic variables (see Table 1).

**Table 1:** Demographic and clinical characteristics at baseline, mean ± SD or *n* (%).

	**EMDR therapy group (*n* = 70)**	**CAU group (*n* = 71)**	***p*-value**
Age (years)	33.5 ± 5.0	34.6 ± 4.6	0.16
Gestational age at randomization (days)	115.6 ± 23.2	121.0 ± 23.1	0.23
Ethnicity*			0.62
Dutch	55 (78.6)	60 (84.5)	
Other, European	7(10.0)	6 (8.5)	
Other, Non-European	6 (8.6)	5 (7.0)	
Mixed	2 (2.9)	0 (0.0)	
Educational level^a^*			0.40
Low	15 (21.5)	9 (12.7)	
Middle	19 (27.1)	25 (35.2)	
High	35 (50.0)	33 (46.5)	
Marital status*			0.47
Co-habiting with partner	60 (85.7)	58 (81.7)	
Partner, not living together	8 (11.4)	5 (7.0)	
No partner	0 (0.0)	3 (5.6)	
Other	1 (1.4)	1 (1.4)	
Parity			0.78
Nulliparous	33 (47.1)	34 (47.8)	
Multiparous	37 (52.9)	37 (52.1)	
Pregnant after fertility treatment*	12 (17.1)	7 (9.9)	0.32
Current use of psychoactive medication*	10 (14.3)	4 (5.6)	0.76
Comorbid disorder			
Mood disorder	10 (14.3)	12 (16.9)	0.67
Anxiety disorder	11 (15.7)	13 (18.3)	0.68
Suicide risk (low)	6 (8.6)	7 (9.9)	0.79
Traumatic experiences^b^	15 (21.4)	11 (15.5)	0.33
Previous childbirth	31 (44.3)	30 (42.3)	
Sexual abuse	3 (4.3)	2 (2.8)	
Physical abuse	2 (2.9)	5 (7.0)	
Work-related	2 (2.9)	1 (1.4)	
Natural disasters, accidents, victims of war	11 (15.7)	8 (11.3)	
Other	2 (2.9)	4 (5.6)	
Previous diagnosis of PTSD*	9 (12.9)	7 (9.9)	0.64
Previous EMDR therapy*	13 (18.6)	9 (12.7)	0.39
Traumatic childbirth	4 (5.7)	4 (5.6)	
Sexual assault	2 (2.9)	0 (0.0)	
Other	7 (10.0)	(7.1)	

*^a^Low: completed high-school (Dutch: MAVO, HAVO, VWO, MBO). Middle: applied sciences (Dutch: HBO). High: completed degree (Dutch: WO).*

*^b^Defined by Life Events Checklist-5 (LEC-5) (44).*

*5 missing (4 CAU, 1 EMDR).

*CAPS, Clinician Administered PTSD Scale; CAU, Care-as-Usual; EMDR, Eye Movement Desensitization and Reprocessing therapy; PCL-5, PTSD Checklist for DSM-5*.

### Safety Measurements

#### Worsening of Symptoms of FoC

A reliable worsening of symptoms of FoC occurred in 6.9% (4/58) in the EMDR group, vs. 13.5% (7/52) in the CAU group, $\chi_{\left(1\right)}^{2}$ = 0.85, *p* = 0.36.

#### Treatment Dropout

Ten out of 70 patients who were randomized to receive EMDR therapy did not start treatment. This was due to logistical or psychosocial problems (*n* = 4), obstetrical reasons (*n* = 2), no show for unknown reasons (*n* = 2), spontaneous remission of symptoms (*n* = 1), or loss of interest (*n* = 1). During EMDR treatment, the dropout rate was 10%: of the 60 women who started therapy, six (10%) discontinued treatment prematurely, all after completing one session. This dropout was caused by obstetric reasons (*n* = 3), two consecutive late cancellations/no show (*n* = 1), and non-response (*n* = 1). Lastly, one woman discontinued therapy since she wanted to avoid thinking of any disturbing image, an essential part of the EMDR protocol. Of the remaining 54 women, *n* = 2 participants completed the therapy in one session (3.7%), *n* = 22 in two sessions (40.7%), and *n* = 30 (55.5%) in three therapy sessions. No significant differences in demographics were found between those completing treatment, dropouts, and those who did not start treatment. The percentage of women included in the final analyses did not differ significantly between groups, with 82.9% (58/70) and 73.2% (52/71) completing the second clinical interview in EMDR and CAU, respectively.

In the email survey that was sent 8–12 weeks postpartum, six (21.4%) of the 28 women who replied from the CAU group reported to have initiated treatment by a psychologist themselves during the pregnancy (exact timing of therapy not reported). Of these six, three women received one (*n* = 1), two (*n* = 1), or three (*n* = 1) sessions of EMDR therapy.

#### Serious Adverse Events

Two serious adverse events (SAEs) occurred during this trial, one in each randomization group. In the EMDR group, one woman with a twin pregnancy gave birth before the fetuses were viable (immature birth), and in the CAU group, an intra-uterine fetal death occurred at 21 weeks of gestation.

#### Suicide Risk

None of the participants developed intermediate or high suicide risk during the trial period, and one woman in the CAU group developed *de novo* low suicide risk. Of the 13 women with low suicide risk at the start of the trial, the low risk remained present in six women (2/6 in the EMDR group and 4/7 in the CAU group), and resolved for seven women (4/6 in the EMDR group and 3/7 in the CAU group).

### Fear of Childbirth

**Figure 2:**
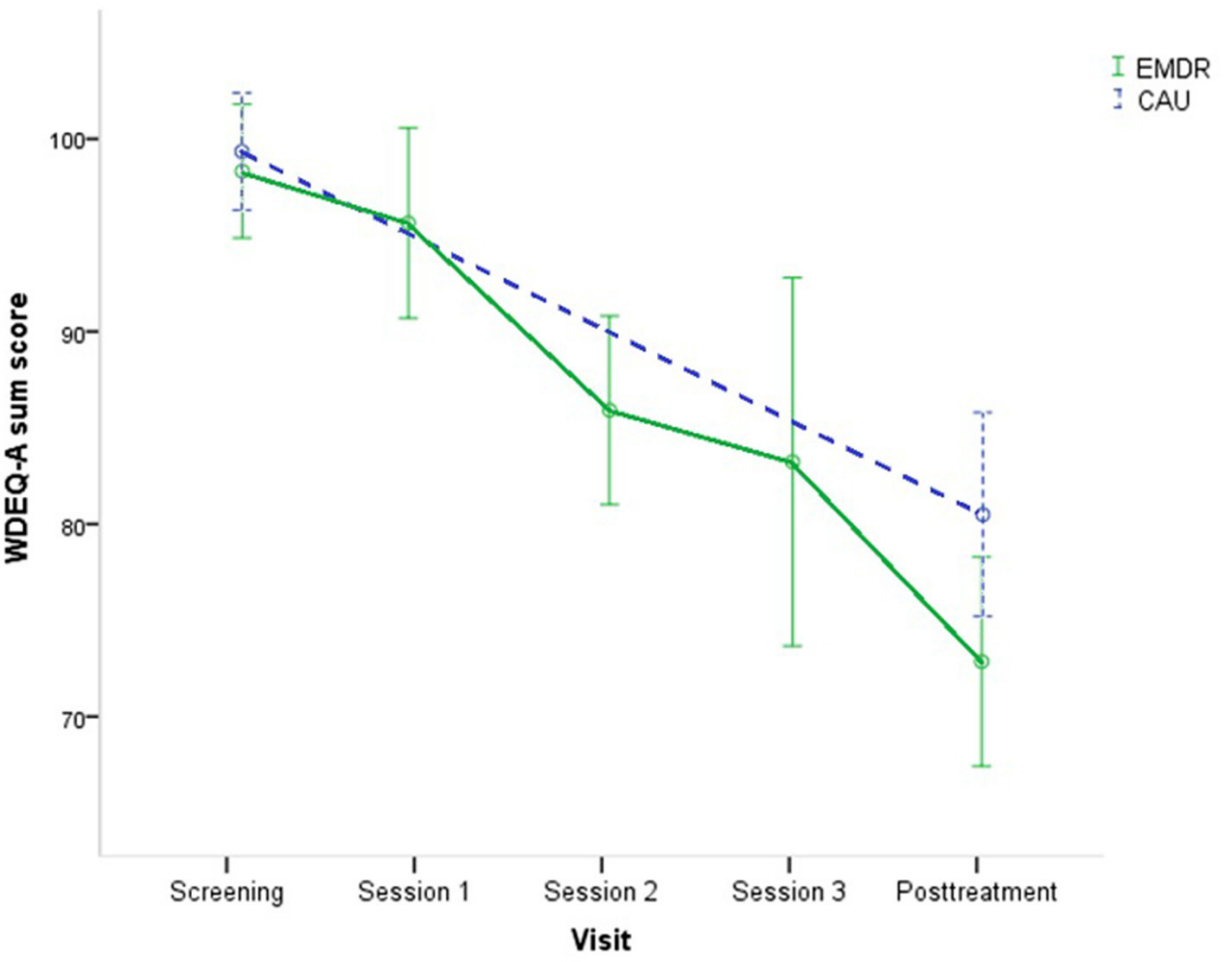
Mean WDEQ-A scores with 95% C.I. at each time point, for EMDR therapy group (*n* = 58) and CAU (*n* = 52) condition. CAU, Care-as-Usual; EMDR; Eye Movement Desensitization and Reprocessing therapy; WDEQ-A, Wijma Delivery Expectancy Questionnaire—version A.

Both groups showed a very large (EMDR therapy, Cohen's *d* 1.36) or large (CAU, Cohen's *d* 0.89) and clinically meaningful reduction of FoC at each time point compared to pre-treatment. Mean WDEQ-A scores at each time point can be seen in Figure 2. Usage of a random intercept significantly improved the model and was included to the LMM analysis. The LMM analysis showed a non-significant decrease in FoC symptom severity in the EMDR therapy group compared to CAU (Table 2), *t*_(2, 67)_ = 0.23, *p* = 0.83, Cohen's *d* = 0.39.

**Table 2:** WDEQ-A scores (mean ± SD) at baseline (screening), sessions, and posttreatment (T1) in the EMDR therapy (*n* = 58) and CAU group (*n* = 52), within-group and between-group effect sizes.

**Groups**	**Measures**					**Within group effect sizes**	**Effect sizes of EMDR vs. CAU**	
	**Screening**	**Session 1**	**Session 2**	**Session 3**	**T1**	**Cohen's *d***	***p*-value**	**Cohen's *d***
EMDR	99.4 (14.6)	96.6 (18.4)	86.8 (17.4)	84.1 (24.2)	73.9 (20.7)	1.36	0.83	0.39
CAU	99.9 (12.9)	x	x	x	81.9 (19.1)	0.89		

*CAU, Care-as-Usual; EMDR, Eye Movement Desensitization and Reprocessing therapy; T1, posttreatment assessment at 32–34 weeks gestational age; WDEQ-A, Wijma Delivery Expectancy Questionnaire—version A*.

In the EMDR therapy group, 86.2% showed reliable symptom improvement on the WDEQ-A, with a mean decrease of 25.6 points (SD = 18.9). In the CAU group, 76.9% showed reliable symptom improvement, with a mean decrease of 17.4 points (*SD* = 19.6) on the WDEQ-A. There was no significant difference in women with a sum score below the WDEQ-A cutoff FoC in the EMDR group (72.4%) compared to the CAU group (59.6%) [$\chi_{\left(1\right)}^{2}$ = 2.01, *p* = 0.17].

## Discussion

The results of this study support our first hypothesis regarding safety, showing that the application of EMDR therapy was not associated with a significant increase of symptoms of FoC, the occurrence of SAEs, increased suicide risk, or a large dropout, when compared to CAU. Conversely, the results were not supportive of our second hypothesis regarding the effectiveness of EMDR therapy, showing that EMDR therapy did not result in a significantly greater FoC symptom reduction compared to CAU.

Although studies with similar data on safety are lacking, the results are in line with a systematic review regarding the effectiveness of EMDR therapy in pregnant women with PTSD (25), which suggests that treatment is likely safe. Furthermore, the results of the current study demonstrate that EMDR therapy resulted in a significant decrease of FoC symptoms, with a very large effect size (Cohen's *d* 1.36). This effect size is comparable with large to very large effect sizes reported in online cognitive behavioral therapy (45), motivational interviewing psychotherapy (16), and haptotherapy (17), and higher compared to psycho-education carried out by midwives (15).

For the finding that no significant difference was found between EMDR therapy and CAU regarding the decrease in FoC, several explanations may be proposed: (1) A considerable number of participants (22.9% of the women randomized for EMDR therapy) did not start therapy or dropped out after only one treatment session, with the most common reasons being logistical or psychosocial problems. Yet, additional analyses (data not shown) comparing only treatment completers to the CAU group on the decrease in FoC could not detect a significant difference between both groups in decrease in FoC. (2) The CAU group showed an unexpected large decrease of FoC. Based on several cross-sectional studies, one would rather assume that FoC would be stable or even aggravate during pregnancy, since delivery is getting closer and inevitable (46–48). In several previous studies, participants in observational arms or CAU conditions were stable or showed an increase in symptoms (18, 47, 49). However, our finding is consistent with the results of another recent Dutch RCT comparing haptotherapy, psycho-education, and CAU (17). The symptom decline in the CAU group suggests natural recovery taking place, and that FoC may be self-limiting in some cases. (3) Part of the women in the CAU group had been treated for their FoC outside the context of this study. At postpartum follow-up, 21.4% of the women who were randomized for CAU stated to have initiated treatment for their fears themselves, half of which received EMDR therapy. However, additional analysis (data not shown) did not show different results. (4) Recruiting for the study may have led to more awareness among participating healthcare providers, resulting in improvement of FoC-informed antenatal care for both treatment groups. (5) For the pregnant women involved, their participation in this study resulted in more and specific attention from professionals for their psychological well-being, with comprehensive psychological interviews being part of this research. The identification and acknowledgment of FoC and the psychological burden that comes with it could have had therapeutical effects in itself (50). A strength of the current study is that our study took place in a real-world clinical setting in both hospital and midwifery practices with a heterogeneous population, thereby enhancing the generalizability of the results. Also, all new pregnant women were invited in a standard way to take part in the screening for FoC, thereby decreasing the risk of selection bias. Conversely, there are also limitations of the present study that need to be mentioned: (1) We did not include a control condition other than CAU. Accordingly, no comparison could be made with a gold standard treatment for FoC since this is not available yet. In the current study, a lack of a (placebo or gold standard) intervention in the control group might have resulted in the relatively high number of women who initiated psychological treatment themselves. Comparing the effectiveness of several treatment conditions including FoC-informed maternity care is an important issue for future research. Besides focusing on efficacy, we suggest future studies to aim to identify risk factors that hinder spontaneous decrease of FoC during pregnancy because this may help to identify women who are likely to benefit most from EMDR therapy. (2) Certain populations were excluded. Women with intermediate or high suicide risk or severe psychotic disorder were excluded because of the low-risk study design. Women currently receiving psychological treatment (15.0% of all women with a positive screening for FoC) were excluded. It is important to include assessment of FoC during any psychological treatment in childbearing women. (3) Women with a gestational age below 8 or above 20 weeks at time of screening were not included to avoid dropout due to non-vital pregnancies and to have sufficient time to plan therapeutic sessions during the remainder of the pregnancy. In future studies, women should be included independent of gestational age, with sample size calculation generously adjusting for rates of dropout and crossover. Lastly, this study was carried out in a high-income country with good access to public health services, as can be seen in the high percentage of women with prior EMDR treatment (Table 1). Most women in our sample were highly educated, cohabiting/married, and Dutch. Although influence of these factors on remission of FoC is unknown (46), it may lead to a lack of generalizability.

In conclusion, the results of this study suggest that EMDR therapy in pregnant women with FoC is safe regarding psychological outcomes, and effective in reducing FoC. However, no significant beneficial effects of EMDR therapy over and above those of CAU were found. Accordingly, although EMDR therapy and CAU may both be viable options to choose for the treatment of pregnant women with FoC, the current study does not justify implementation of EMDR therapy as an additional treatment in this particular setting. Clearly, while replication of our findings is important, for future research, it is important to determine whether there may be certain subgroups of patients for which trauma-focused treatment does have added value, for example, in the case that the offered standard care does not automatically lead to alleviation of fear and patients are at risk having to endure childbirth with great fear. Hence, further studies are needed to indicate which subgroups have low chance at spontaneous decrease of FoC and might benefit most from EMDR therapy.

## Data Availability Statement

The data that support the findings are available from the corresponding author, M. G. van Pampus, upon reasonable request.

## Ethics Statement

The studies involving human participants were reviewed and approved by Medical Research Ethics Committee. The patients/participants provided their written informed consent to participate in this study.

## Author Contributions

MB drafted the paper under supervision of AdJ and MvP. All authors have made substantial contributions to the concept of this study and were involved in critically revising the manuscript and accepted the final manuscript.

## Funding

This work was supported by grants from the Stichting EMDR Nederland (Dutch EMDR Association), Fonds Gezond Geboren, Stichting Teaching Hospital OLVG, and Stichting Wetenschap OLVG, all awarded to the principal investigator MvP.

## Conflict of Interest

Author AdJ has been a board member of the Dutch EMDR Association and EMDR Europe Association, and receives fees for courses and books about trauma and EMDR. The remaining authors declare that the research was conducted in the absence of any commercial or financial relationships that could be construed as a potential conflict of interest.

## Publisher's Note

All claims expressed in this article are solely those of the authors and do not necessarily represent those of their affiliated organizations, or those of the publisher, the editors and the reviewers. Any product that may be evaluated in this article, or claim that may be made by its manufacturer, is not guaranteed or endorsed by the publisher.
